# Frequency of Gestational Diabetes Mellitus Reappearance or Absence during the Second Pregnancy of Women Treated at Mayo Clinic between 2013 and 2018

**DOI:** 10.1155/2019/9583927

**Published:** 2019-11-22

**Authors:** Elizabeth Ann L. Enninga, Aoife M. Egan, Layan Alrahmani, Alexey A. Leontovich, Rodrigo Ruano, Michael P. Sarras

**Affiliations:** ^1^Department of Obstetrics and Gynecology, Mayo Clinic, 200 First Street SW, Rochester, MN, USA; ^2^Department of Endocrinology, Mayo Clinic, 200 First Street SW, Rochester, MN, USA; ^3^Department of Health Science Research, Mayo Clinic, 200 First Street SW, Rochester, MN, USA; ^4^Department of Cell Biology and Anatomy, Rosalind Franklin University of Medicine and Science, 3333 Green Bay Road, N. Chicago, IL, USA

## Abstract

The Center for Disease Control and Prevention ranks diabetes mellitus (DM) as the seventh leading cause of death in the USA. The most prevalent forms of DM include Type 2 DM, Type 1 DM, and gestational diabetes mellitus (GDM). While the acute problem of diabetic hyperglycemia can be clinically managed through dietary control and lifestyle changes or pharmacological intervention with oral medications or insulin, long-term complications of the disease are associated with significant morbidity and mortality. These long-term complications involve nearly all organ systems of the body and share common pathologies associated with endothelial cell abnormalities. To better understand the molecular mechanisms underlying DM as related to future long-term complications following hyperglycemia, we have undertaken a study to determine the frequency that GDM did or did not occur in the second pregnancy of women who experienced GDM in their first pregnancy between 2013 and 2018 at Mayo Clinic, Rochester, MN. Within the five-year period of the study, the results indicate that 7,330 women received obstetrical care for pregnancy during the study period. Of these, 150 developed GDM in their first pregnancy and of these, 42 (28%) had a second pregnancy. Of these 42 women, 20 again developed GDM and 22 did not develop GDM in their second pregnancy within the study period. Following the occurrence of GDM in the first pregnancy, the study (1) established the number of women with and without GDM in the second pregnancy and (2) confirmed the feasibility to study diabetic metabolic memory using maternal placental tissue from GDM women. These studies represent Phase I of a larger research project whose goal is to analyze epigenetic mechanisms underlying true diabetic metabolic memory using endothelial cells isolated from the maternal placenta of women with and without GDM as described in this article.

## 1. Introduction

The Center for Disease Control and Prevention now ranks diabetes mellitus (DM) as the seventh leading cause of death in the USA with some 80,000 fatalities a year [1]. The total number of individuals affected by the disease in the USA is approximately 30 million with global numbers approaching 642 million by 2040 [2, 3]. Diabetes is classified as a disease of metabolic dysregulation of feedback systems that regulate glucose homeostasis [4]. The disease has a number of prevalent forms such as Type 1 DM (T1DM), Type 2 DM (T2DM), and gestational diabetes mellitus (GDM), as well as a number of minor forms that also involve induced hyperglycemia [5]. T1DM typically has an autoimmune etiology where pancreatic beta cells are targeted, T2DM involves environmental and lifestyle effects on ligand-receptor systems, while GDM is a pregnancy-associated form of diabetes that reverses following birth [5–8]. In regard to T1DM and T2DM, evidence indicates that both involve selective genes associated with risk for the disease which leads eventually to acute hyperglycemia [4, 9]. While the acute problem of hyperglycemia can be clinically managed through dietary control and lifestyle changes or pharmacological intervention with oral hypoglycemic medications or insulin [4], the more severe aspects of the disease include mortality resulting from long-term complications [4] such as cardiovascular disease involving both microvascular and macrovascular components [10–13], retinopathy, nephropathy, neuropathy, and impaired wound healing [14–16]. These long-term complications involve nearly all organ systems of the body and share common pathologies associated with endothelial cell abnormalities. Endothelial cell dysfunction in DM [17] takes on different forms to include (1) altered compliance [18], (2) acquired vascular flow abnormalities [19], and (3) altered blood vessel growth through both angiogenesis [10, 12, 20] and neovascularization [13, 21–24].

To better understand the molecular mechanisms underlying gestational diabetes mellitus (GDM) as related to future long-term complications following hyperglycemia, we have undertaken a study at Mayo Clinic (Rochester, MN) to determine the frequency that GDM did or did not occur in the second pregnancy of women who experienced GDM in their first pregnancy between 2013 and 2018. The reasons for these specific parameters will be explained when we discuss the long-term goals of our ongoing research in the Discussion section.

## 2. Materials and Methods

The Mayo Clinic Investigational Review Board approved all methods related to this study (IRB #: 17-009957). This is a retrospective cohort study that included pregnant women who had their first pregnancy and were diagnosed with gestational diabetes between January 1st, 2013 and December 31st, 2017. The cohort was identified by searching the electronic medical records by ICD-9 and ICD-10 codes 648.8, O24.410, O24.414, O24.419, O24.420, O24.424, and O24.343. All subjects were between 18 and 35 years of age at their first pregnancy, were having singleton pregnancies, and did not have a diabetes diagnosis prior to pregnancy (HgA1c < 6.5%). This cohort was then followed until the end of 2018 to record whether or not they had a second pregnancy diagnosed with gestational diabetes.

We utilized the American College of Obstetricians and Gynecologists (ACOG) Clinical Management Practice Guidelines [25] to diagnose gestational diabetes. All subjects underwent 1-hour oral glucose testing between 24 and 28 weeks at Mayo Clinic. Those with a result of <140 mg/dL were not diagnosed with gestational diabetes while those with a result of ≥140 mg/dL were required to do a 3-hour oral glucose test. Normal values for 3-hour glucose testing were fasting ≤ 95 mg/dL, 1 hour ≤ 180 mg/dL, 2 hour ≤ 155 mg/dL, and 3 hour ≤ 140 mg/dL. If the subject had two abnormal results during their 3-hour test, they were diagnosed with gestational diabetes and counseled on proper glucose management. Preliminary diagnosis was made by the examining physician, all cases were abstracted by three residents, and data were collected in a RedCap database. Validation of data collection was completed by the study statistician using random sampling. A broad spectrum of clinical variables were collected for each pregnancy, but for the Results we listed the following: age, body mass index (BMI), race, smoking history, family DM history, glucose levels, and clinical management of GDM throughout pregnancy.

## 3. Results

**Figure 1:**
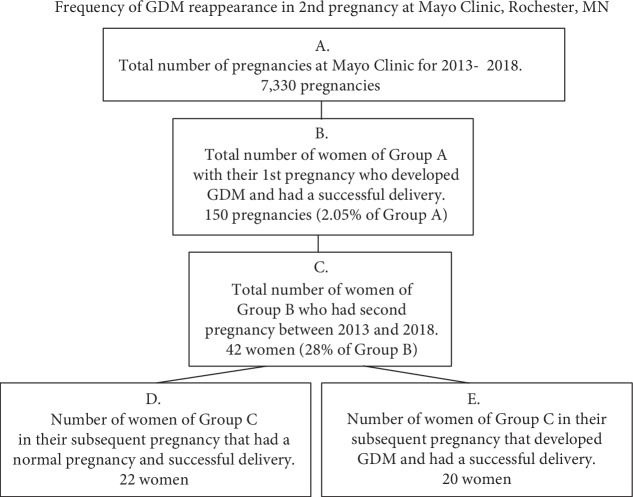
Diagrammatic breakdown of the results of retrograde analysis of women developing GDM in the first and second pregnancies at Mayo Clinic, between 2013 and 2018. As indicated, women who developed GDM in their first pregnancy (Group B) were followed to determine the occurrence (Group E) or lack of occurrence of GDM (Group D) in their second pregnancy in the time frame of the study.

The overall results from a review of clinical records for the five-year period between January 1, 2013 and December 31, 2018 found that 7,330 women received their obstetrical care at Mayo Clinic (Rochester, MN) for pregnancy (Figure 1).

**Figure 2:**
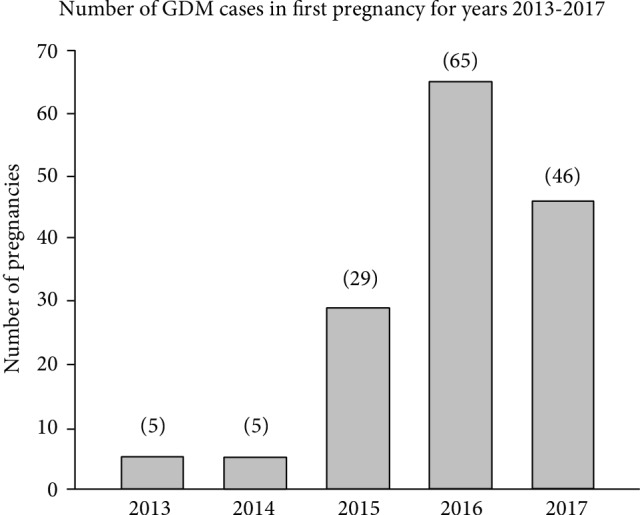
Numerical breakdown of the number of cases in which women developed GDM in their first pregnancy between the years 2013 and 2018 at Mayo Clinic, Rochester, MN. It should be noted that the increase in GDM cases in the final three years of the study can be explained by a change in screening methods for GDM. This indicates that a greater number of patients falling within Groups B, C, D, and E of Figure 1 will be available in future studies carried out over a five-year period.

Of these 7,330 women, 150 were diagnosed with GDM in their first pregnancy (Group B, Figure 1). As shown in Figure 2, between 2013 and 2018, there was a significant increase in the number of GDM cases diagnosed in the third year of the study. Of these 150 women (Group B), 42 were found to have a second pregnancy (Group C) within the five-year period of the review as also shown in Figure 1. The 42 women of Group C were further divided into two subgroups, with 20 again developing GDM (Group E) and 22 not developing GDM (Group D) in their second pregnancy (Figure 1). Figure 2 shows the number of GDM cases of women with GDM in the first pregnancy during the five-year study period.

Clinical parameters that were studied in Groups B, D, and E are shown in Tables 1 and 2. In Table 1, the clinical parameters included age, weight, BMI, and glucose levels from 1-, 2-, 3-hour and postparturition tests for those women who had fasting glucose levels greater than 140 mg/dL. Data is presented as means with standard deviation values.

Additional clinical parameters are shown in Table 2 that depicts race/ethnicity, smoking history, occurrence of diabetes mellitus in the family, and clinical management procedures for women diagnosed with GDM.

## 4. Discussion

The data presented in this article represent initial studies pertaining to a larger research project ongoing in our laboratory. The goal of that larger project is to analyze epigenetic mechanisms underlying true diabetic metabolic memory using tissues obtained from patients being treated at Mayo Clinic, Rochester, MN. The problems associated with studying metabolic memory (MM) in the human diabetic condition stems from the basic nature of diabetes. From a clinical standpoint, evidence from both the laboratory [26–32] and large scale human trials [33–37] has revealed that complications from the onset of hyperglycemia progress unimpeded via the phenomenon of MM even when glycemic control is pharmaceutically achieved [33–37]. This applies to both T1DM and T2DM. The underlying molecular mechanisms of hyperglycemic complications and MM may include (1) the involvement of excess reactive oxygen species, (ROS) (2) the involvement of advanced glycation end products (AGE), and (3) alterations in tissue-wide gene expression patterns [4]. The production of ROS and AGE by hyperglycemia is a continual process throughout the life of those with diabetes. Even those whose glycemic levels are well controlled, all with this disease have episodic variations in their glucose and therefore episodic hyperglycemia continually occurs. This complicates the study of MM because the variables of ROS and AGE production are always present and therefore a “pure metabolic memory state” in which the mechanisms of MM can be studied in a state of euglycemia never exists.

The heritable nature of MM [38, 39] suggests a role for the epigenome. The epigenome is comprised of all chromatin-modifying processes including DNA methylation and histone modifications allowing cells/organisms to quickly respond to changing environmental stimuli [40–42]. These processes not only allow for quick adaptation but also confer the ability of the cell to “memorize” these encounters [40–42]. As indicated, alterations in blood vessel growth affects a wide spectrum of organs/tissues in DM thereby causing systemic problems [2]. The underlying molecular mechanism(s) of MM have been examined via both animal model approaches and *in vitro*-based studies [26–32]. These studies establish that the initial hyperglycemia results in permanent aberrant gene expression in DM target tissues (e.g., cardiovascular system, kidney, retina, skin as related to wound healing, and impaired blood vessel growth such as seen in the wound healing process). In this regard, epigenetic research pertaining to DM has been conducted regarding histone modifications [43, 44], microRNA mechanisms [45, 46], and to a lesser degree, hyperglycemia-induced persistent DNA methylation changes [47]; however, epigenetic studies on pure MM (in the absence of hyperglycemia) have only been achieved in specialized animal model studies [48].

Based on an animal model developed in our laboratory [30], we have previously reported that hyperglycemia induces aberrant DNA methylation with concomitant altered gene expression patterns that correlate with persistent diabetic complications. In brief overview, the Zebrafish allows for ablation of pancreatic beta cells to induce a diabetic hyperglycemic state followed by regeneration of those pancreatic beta cells. While in a hyperglycemic state, tissue dysfunction as observed in the long-term diabetic condition (retinal tissue, renal tissue, impaired wound healing, and impaired angiogenesis) is observed in the model [30, 49]. With beta cell regeneration, there is a return to normal systemic glycemic control (euglycemia) and therefore a “true metabolic memory” state was induced with the absence of any hyperglycemia. Although glucose control returned to normal, all the tissue dysfunction observed during the hyperglycemic diabetic state continued to be observed. This suggested that the temporary hyperglycemic state was “remembered” once euglycemia was reestablished. Genomic analysis indicated that changes in DNA methylation occurred during the DM state and this was accompanied with altered gene expression patterns. Each tissue had its own mRNA expression profile changes that reflected the tissue studied. The DNA methylation status of many loci were permanently altered in regard to their methylated status, and when this data was viewed within the context of global gene expression (via microarray analysis), a correlation of permanent CpG island DNA methylation changes and altered expression was observed [50]. Persistent hyperglycemia-induced impaired tissue regeneration correlated directly with aberrant DNA methylation and metabolic memory gene expression changes [30]. A similar molecular mechanism may exist in human DM patients as related to the pathologies observed in the endothelial cell. In this regard, the current GDM project is an approach to study diabetes and metabolic memory in adult human endothelial cells exposed to hyperglycemia.

To expand the animal model studies described above to the human DM condition, we have devised a strategy to develop human studies that allow analysis of a “true metabolic memory” state in human cells, specifically, human adult endothelial cells. Briefly, those studies entail analysis of isolated endothelial cells of the maternal placenta (decidua basalis) from women with and without gestational diabetes mellitus (GDM). The paradigm will obtain the maternal placenta from the same women in their first and subsequent pregnancies and analyze the isolated endothelial cells of these women. Four groups will be studied to include (1) women without GDM, (2) women with GDM in their first pregnancy (Group B in Figure 1), (3) the women of Group B who in their subsequent pregnancy (3) have GDM again (Group E), or (4) do not develop GDM (Group D). It is important to note that spiral arteries of the maternal placenta are derived from the uterine wall tissue; therefore, women of Group B have systemic hyperglycemia which exposes the endothelial cells of their uterine wall to hyperglycemic conditions. The same women studied in Group B will be studied in Groups D or E. Therefore, a given woman of Group D would have had their uterine wall endothelial cells exposed to hyperglycemic conditions in their first pregnancy and the spiral artery endothelial cells of the second pregnancy would show any changes if they are retained. Women of Group D therefore represent a “true metabolic memory” condition because the women in Groups D and E will have normal glycemic control between their pregnancies (no episodic hyperglycemic episodes). The glucose levels postparturition for women with GDM indicate that normal glucose levels returned following birth (Table 1). This validates the underlying premise of the research strategy that utilizes women without GDM in their second pregnancy following a first pregnancy with GDM thus allowing analysis of a “true metabolic memory” state for these women (systemic hypoglycemia followed by normal glycemic control). This approach requires that we study women of Group B who have no history of diabetes and no previous pregnancies with GDM. As mentioned previously, metabolic memory is a phenomenon in which the initial hyperglycemic changes are “remembered over time.” This occurs naturally in GDM, allowing us to use the temporary hyperglycemic condition that exists in GDM to study metabolic memory in those women who do not experience GDM in their second pregnancy. To our knowledge, this is the only approach that allows the study of human adult cells in a “true metabolic memory” condition (endothelial cells of the decidual basalis of the maternal placental) and we will use this strategy to analyze epigenetic differences between control and GDM groups.

This manuscript represents the first phase in the development of this human study strategy and establishes that through the Mayo Clinic, Rochester, MN; sufficient numbers of women fall into all groups of Figure 1 (to include control groups) to achieve statistically significant results to enable power calculation for epigenetic analyses.

## 5. Conclusions

Following the occurrence of GDM in the first pregnancy, the study (1) established the number of women with and without GDM in the second pregnancy and (2) confirmed the feasibility to study diabetic metabolic memory using maternal placental tissue from GDM women. The data indicated that statistically significant results to enable power calculation for epigenetic analyses could be achieved. These studies represent phase I of a larger research project whose goal is to analyze the role of DNA methylation in the development of true diabetic metabolic memory using endothelial cells isolated from the maternal placenta of women with and without GDM as described in this article.

## Figures and Tables

**Table 1:** Clinical parameters for data analysis 1.

Clinical parameter	1st pregnancy w/ GDM (*N* = 150)	2nd pregnancy w/ GDM (*N* = 22)	2nd pregnancy w/o GDM (*N* = 20)
Age	Range 19-43 years Mean 28.47 ± 4.17 SD	Range 24-39 years Mean 31.30 ± 3.91 SD	Range 21-32 years Mean 28 ± 3.95 SD
Weight	Mean 79.79 kg ±24.88 SD	Mean 81.94 kg ±17.41	Mean 81.96 kg ±25.23
BMI (body mass index)	Mean 29.67 kg/m^2^ ±8.59 SD	Mean 31.07 kg/m^2^ ±6.53	Mean 30.47 kg/m^2^ ±8.91
1 hr glucose level^∗^	Mean of 189.05 mg/dL ±23.67 SD	Mean of 203.09 mg/dL ±21.16 SD	Normal treatment procedures for pregnancy
2 hr glucose level^∗∗^	Mean of 178.83 mg/dL ±24.00 SD	Mean of 199.55 mg/dL ±36.85 SD	Normal treatment procedures for pregnancy
3 hr glucose level^∗∗∗^	Mean of 130.72 mg/dL ±34.32 SD	Mean of 130.91 mg/dL ±24.27 SD	Normal treatment procedures for pregnancy
Postparturition^∗∗∗∗^ glucose levels	Mean of 108.47 mg/dL ±33.05 SD	Mean of 99.77 mg/dL ±13.02 SD	Normal treatment procedures for postpregnancy

Mayo Clinic basis for a GDM diagnosis is as follows: Fasting glucose > 140 mg/dL requires 3 consecutive glucose level tests. If 2 of 3 tests are greater than normal, the woman is diagnosed with GDM. ^∗^Glucose level of less than 180 mg/dL is considered normal. ^∗∗^Glucose level of less than 155 mg/dL is considered normal. ^∗∗∗^Glucose level of less than 140 mg/dL is considered normal. ^∗∗∗∗^Glucose level tested to insure normal glucose levels returned post-GDM.

**Table 2:** Clinical parameters for data analysis 2.

Clinical parameter	1st pregnancy w/ GDM (*N* = 150)		2nd pregnancy w/ GDM (*N* = 20)		2nd pregnancy w/o GDM (*N* = 22)		
Race/ethnicity	White (4 Hispanic of 113 White)	113	White (1 Hispanic of 18 White)	18	White (0 Hispanic of 20 White)	20
	American Indian	1	American Indian	0	American Indian	0
	Alaska Native, Native Hawaiian, or other Pacific Islander	1	Alaska Native, Native Hawaiian, or other Pacific Islander	0	Alaska Native, Native Hawaiian, or other Pacific Islander	0
	Asian	20	Asian	1	Asian	0
	Black or African Am.	10	Black or African Am.	0	Black or African Am.	2
	Multiracial	2	Multiracial	0	Multiracial	0
	Undocumented	3	Undocumented	1	Undocumented	0

Smoking	Never smoked	111	Never smoked	17	Never smoked	18
	Quit > 1 yr	5	Quit > 1 yr	2	Quit > 1 yr	1
	Quit during this pregnancy 12	12	Quit during this pregnancy 0	0	Quit during this pregnancy	0
	Currently smoking	14	Currently smoking	1	Currently smoking	2
	Not documented	8	Not documented	0	Not documented	1

DM in family	DM in family (36 out of 150)		DM in family (0 out of 20)		DM in family (6 out of 22)	

GDM clinical management	Diet and lifestyle	97^∗^	Diet and lifestyle	10	Normal treatment procedures for pregnancy	
	Glyburide	45	Glyburide	6		
	Insulin	8	Insulin	3		
	Other	0	Other	1		

^∗^105 women began as diet and lifestyle treatment, but 8 were changed to glyburide treatment later in their pregnancy.

## Data Availability

All data for this study are available via Mayo's RedCap system which can be accessed via email communication with the first author for an Excel Spreadsheet.
